# Implementation of project management methodologies in microbiology research laboratories

**DOI:** 10.1099/acmi.0.001032.v3

**Published:** 2025-08-19

**Authors:** Piotr R. Stempinski, Heather M. Lamb, Jiaqi Qian

**Affiliations:** 1 W. Harry Feinstone Department of Molecular Microbiology and Immunology, Johns Hopkins Bloomberg School of Public Health, Baltimore, MD 21205, USA; 2 Department of Commerce Operations, Spectrum Brands, Middleton, WI 53562, USA

**Keywords:** leadership development, project management, research design, research management, research methodology

## Abstract

The increasing complexity and collaborative nature of scientific research projects underscore the need to implement project management practices to manage resources and funding, ensure data quality and prevent delays in project progress. Here, we introduce three major project management methodologies, including agile, waterfall and hybrid approaches, and explore their suitability for biological and microbiological research laboratories. Variables that may influence choosing an appropriate strategy for managing projects are considered, including the size and experience of a research group. In the following article, we provide an overview of the five major stages of project planning and execution, focusing on implementing each of the discussed strategies in the research laboratory. Furthermore, we discuss the composition of the research team and outline the responsibilities assigned to each team member based on their role in the project. This paper highlights potential risks and challenges that may negatively impact research progress, underscoring the need for proper project planning. Applying proper project management methodologies is often neglected in academic research, leading to serious delays and waste of valuable resources.

## Data Summary

 No new data have been generated in the process of creating this article.

## Introduction

Hypothesis-driven scientific research is induced by the implementation of the scientific method, often instilled in young children in the primary school science curriculum. While the scientific method provides a framework from which individual scientists explore various aspects of the natural world from an experimental level, management approaches and personal leadership strategies are required for successfully and efficiently implementing a scientific research programme. As an essential component of successful research in biological and microbiological laboratories, project management methodologies integrate the scientific method with team/personnel management to efficiently work toward a common research objective [1]. Applying proper management techniques helps to prepare an organized framework for strategizing and overseeing project progress, guaranteeing adequate fiscal and technological support while adhering to scheduled deadlines. In addition, effective implementation of project management strategies drives laboratory managers to delineate clear expectations and responsibilities, empowering all team members to take collective ownership of the project [2]. Over the past years, project management strategies have become essential in biological and biomedical research laboratories due to escalating intricacy and collaborative nature of research.

Project management is the process of organizing, planning and administering resources to achieve previously established goals and objectives within a specific time frame [3,5]. Implementing project management elements in research laboratories can significantly improve research outcomes by breaking down complex projects into smaller, more manageable stages, identifying and securing resources, creating a detailed project schedule and communicating regularly with team members [6,8]. The roles and responsibilities of research team leaders have evolved considerably during the past decades, with increasing requirements towards quality, ethics and reproducibility of the experiments [9]. The integration of project management can enhance collective efforts among members of a research team, while also identifying and controlling any potential hazards or problems that may appear in the course of project progress [10]. Project leaders can also enhance efficiency and productivity in research laboratories by streamlining processes, dividing tasks among team members and reducing work overlap [11]. Implementing project management strategies in laboratories can lead to better research outcomes, more productive laboratory environments and increased safety, drastically limiting the complications associated with work with biological, especially microbial samples [12]. It is a vital tool to increase the chances that research projects are completed successfully, within the predicted budget, and without delay while achieving the established outcomes and satisfaction of the research team members [6,13, 14]. A growing emphasis on open scientific research, encouraging the free exchange of information and resources, underscores the need for project management strategies to enhance transparency, accountability, collaboration and efficiency.

This paper aims to present the adaptation of project management methodologies in the organization of research laboratories, including a selection of approaches such as agile, waterfall and hybrid methods. It will also examine the potential challenges and risk factors that can lead to project failure in a laboratory environment and how proper project planning and management can help mitigate these risks. Ultimately, this paper will provide recommendations for implementing project management practices in the organization of microbiology research.

## Project management approaches to scientific project development

Given the very complex and sensitive nature of biological and microbial samples, proper planning is essential at every stage of a microbiology-focused research project. Multiple environmental and technical factors, including microbial viability, proper storage, correct identification of desired phenotypes, incubation periods, risk of contamination and biosafety compliance, introduce several variables that may impact project timelines and final outcomes. Microbiological work often involves non-linear progression and precisely controlled experimental conditions, making planning, flexibility and adaptability critical. Agile and waterfall are two popular and easy-to-set-up project management methodologies utilized in research laboratories to enhance productivity and increase the likelihood of project success [15]. Additionally, a hybrid approach combines elements of both methodologies and capitalizes on the strengths of each method. Understanding the differences and selecting the most optimal strategy for project design and execution can benefit research progress.

### Waterfall approach

**Fig. 1.**
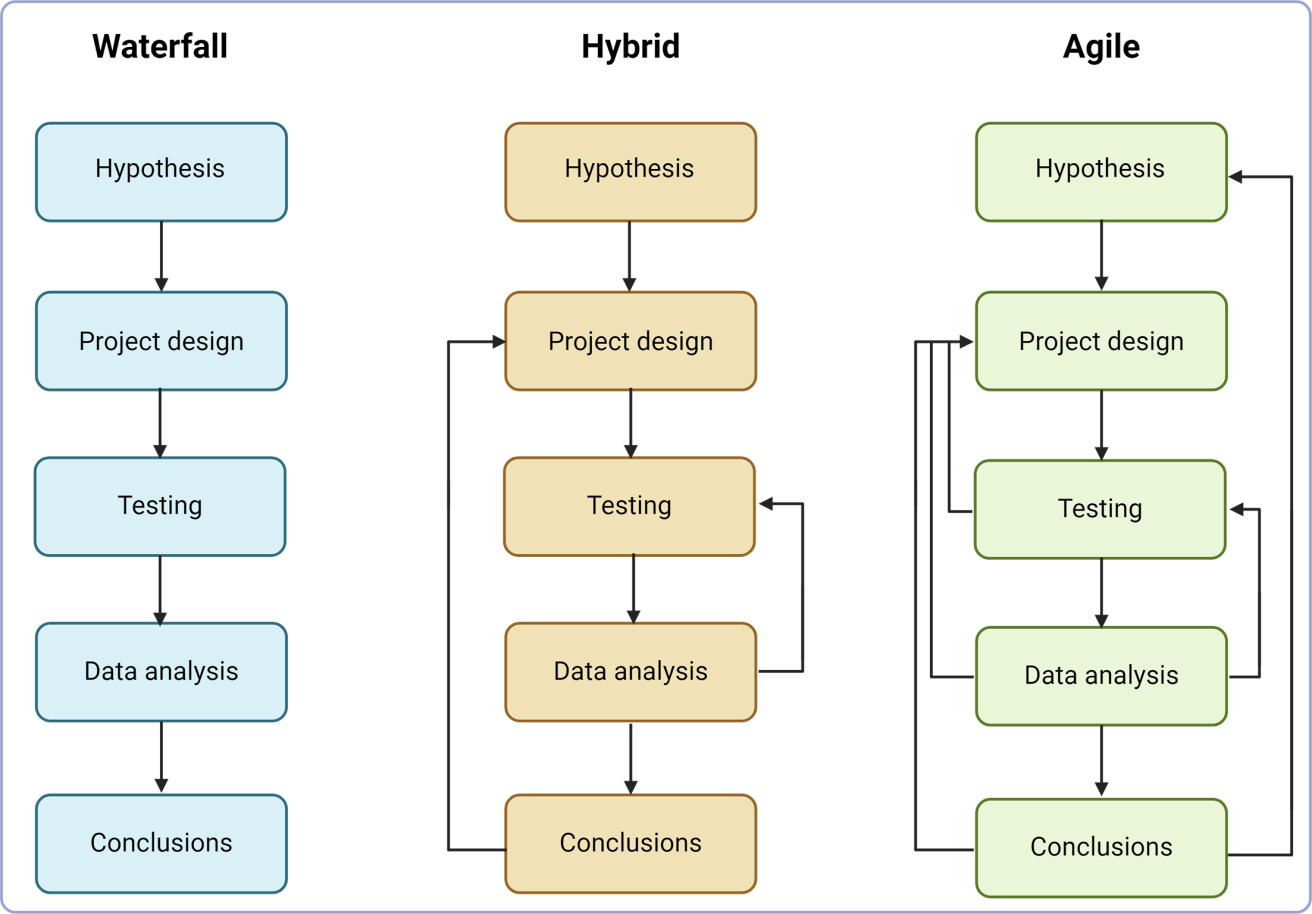
A flow chart visualizing three laboratory project management approaches. Depending on the nature of the project, strategies may be selected that contain well-established and expected milestones and phases (waterfall approach) or dynamic and adaptable based on recurring discoveries (agile approach).

The project management method known as the waterfall methodology focuses on defining and executing a systematic and step-by-step approach (Fig. 1). The waterfall approach is a linear project management methodology commonly used in software development and adaptable to STEM research laboratories, where detailed planning helps provide experimental accuracy, safety and data reproducibility [16].

The distinctive feature of this approach establishes a systematic progression of steps that are followed in order, completing each segment before proceeding to the next phase. The process requires one to accomplish every step sequentially from commencement until conclusion [16,18]. By focusing on detailed planning, sample preparation, incubation, analysis and reporting, the linear structure of the waterfall methodology can reduce the risk of errors or missteps, which increases laboratory workflows and the chance of project success. The waterfall approach is particularly useful for research projects with well-defined goals, methodologies and a clear path to completion. One of the biggest advantages of the waterfall approach in laboratory project management is its focus on comprehensive planning and detailed documentation [16]. The coherent and systematic structure of the waterfall methodology necessitates detailed outlining and recording for each stage to avoid errors, as well as guaranteed reproducibility of data [19]. Moreover, meticulous documentation is essential for subsequent research endeavours or publications, as it provides a clear record of the project’s methodology and results [17,20]. Comprehensive documentation further supports future research initiatives and facilitates the preparation of publications, offering a transparent account of the project’s procedures and outcomes [21]. Another advantage of the waterfall approach is its simplicity and coherence. The waterfall approach is particularly advantageous in academic research laboratories where projects regularly include inexperienced students or early-career research scientists. Core laboratory facilities may also benefit from the waterfall approach. The systematic methodology is especially helpful to understand and follow, making it particularly useful for teams with little experience. Additionally, separating the project tasks into smaller stages ensures that research team members are aware of their precise roles and responsibilities and are able to work efficiently, utilize their best abilities and improve overall workplace organization. By breaking the project into distinct, smaller phases, teams can concentrate on finishing each stage before moving on to the next, ensuring that the project progresses without problems and achieves all its goals. This system is especially well-suited for microbiological research involving the analysis of large numbers of samples, where thorough organization and detailed categorization of tested material are critical for the accuracy of data.

### Agile approach

Microbiological research involves working with dynamic biological systems, where microbial growth patterns or interactions could result in unpredictable results. Agile methodology is an increasingly popular project management approach in research laboratories by emphasizing flexibility, adaptability and collaboration and is particularly useful for projects with constantly evolving outcomes or where experimentation is required to determine the next step of the project [15,22, 23]. By breaking down projects into smaller, iterative cycles, the agile methodology allows for frequent feedback and adjustment throughout all phases of the project, enabling teams to respond quickly to changes and adjust their approach as needed in light of new findings (Fig. 1). This approach can improve productivity and increase the chances of success for projects that are highly dependent on experimental outcomes, such as drug and vaccine development, or genetic engineering (18). Utilizing agile methodology in laboratory project management grants a substantial benefit, namely the facilitation of experimentation and innovation. By breaking the project into smaller, more manageable cycles, teams can experiment with different approaches and improve their methods according to the incoming findings [15,22]. This allows researchers to test and redesign their ideas, leading to more innovative and practical solutions to complicated problems. Additionally, the frequent feedback and adjustment process can help prevent errors and missteps, reducing the risk of project failure. Another advantage of the agile approach is its emphasis on collaboration, teamwork and creativity. Agile methodology encourages partnerships between team members, which is beneficial to the exchange of innovative concepts and solutions needed to accomplish project objectives [15]. This leads to a more effective and efficient workflow and a more cohesive team, ultimately leading to better research outcomes. Due to the flexible and constantly evolving nature of the project, the agile approach requires the project lead to possess effective communication skills to ensure that all team members are familiar with the newest changes in research design [23]. While the agile methodology is amenable to an exploratory approach to research projects, goals and milestones are often less well-defined and, thus, may not be advantageous for early-stage trainees.

### Hybrid approach

In microbiological research, where some procedures require organized, sequential execution while others demand flexibility to adapt to experimental outcomes, a hybrid approach provides elements of both agile and waterfall methodologies and can be used to capitalize on the strengths of each approach [16]. For example, a hybrid approach might involve breaking down a project into smaller, iterative cycles (agile) but also include a scheduled planning and execution phase for each cycle (waterfall) [20,24]. Facilitating a well-ordered and successful project execution can be guaranteed by adhering to structured planning principles (Fig. 1). Such an approach allows for adjustments when unexpected circumstantial changes arise, preserving the necessary flexibility essential in these types of endeavours [24]. The flexibility of the hybrid approach in laboratory project management is a substantial advantage. This methodology of merging two or more techniques empowers researchers to modify their projects towards varying directions as required, without constraining themselves within pre-established boundaries while still maintaining complete control over specific aspects where necessary [25]. The hybrid approach enables teams to adjust their project design to fit the specific needs of the project, which may be based on ongoing experimental results. For example, the team may choose to use the waterfall approach for certain phases of the project that have well-defined goals and requirements but switch to the agile methodology for phases that require more flexibility and exploratory experimentation [26,27]. Another advantage of the hybrid approach is its ability to support innovation and creativity, which is essential for the research and development of novel laboratory techniques [20]. The elements of agile methodology used in the hybrid approach encourage experimentation and frequent feedback, allowing teams to test, analyse and refine their ideas and approaches while improving the quality of experiments [28]. Simultaneously, the step-by-step methodology adapted from the waterfall approach offers an organized framework and direction, ensuring the continuation of research progress and achievement of its objectives. The flexibility of the approach allows teams to adjust their approach as needed, enabling them to respond quickly and effectively to changes, without compromising the progress of the project [28].

## Stages of project development and implementation

To increase the chances of success, each project plan is separated into five phases and followed according to the selected management approach (Fig. 1).

### Hypothesis

This phase involves gathering essential information about the tested problem, including performing literature searches and evaluating preliminary experimental findings and available resources [29,30]. Based on this information, a statement encompassing the scientific research question and predicted outcome should be articulated and communicated to team members [31]. Before transitioning to the next phase, the scope of the project should be clearly defined to meet a specific endpoint (such as a completed manuscript or grant submission) [32,33]. The team leader/principal investigator is responsible for establishing milestones in the project plan. In addition, the team should select the preferred form of communication and data storage [31].

### Project design

Based on the selected hypothesis, the project lead/primary investigator identifies a project manager and cooperatively creates a comprehensive experimental design that includes detailed project plans [29]. Laboratory personnel are assigned tasks congruent with the articulated goals and expertise. As the experimental strategies are designed, personnel training, biosafety and/or IRB approvals, equipment, necessary supplies, reagents and other resources should be acquired. Risk assessments should also identify potential hazards and preventative measures to implement the project safely. Outside collaborations should be established, with clearly defined roles and expectations outlined. Based on the assigned duties, project members must clearly understand the project scope and goals [31]. Discussions regarding authorship should be initiated, and a strategy for determining author order needs to be communicated. An often-overlooked component of the project design phase entails determining sample size and performing power calculations, which is critical for achieving the statistical significance needed to support the hypothesis. In this phase, the team should also establish the frequency and length of the project meetings. Additionally, at this stage, the team should be able to identify and forecast major challenges that may occur during the realization of the project and set up countermeasures to prevent money and time losses. It is the project leader’s responsibility to maintain a clearly defined scope and research focus, which is essential for sustaining the team’s motivation [33].

Designing an effective timeline is essential for the success of scientific projects, providing a clear structure, improving time management, facilitating communication and allowing for progress monitoring. A well-designed timeline organizes the project into manageable phases and tasks, ensuring each step is planned and assigned to the right person. Deadlines for important milestones are identified to help manage time and resources efficiently, avoiding significant delays. Additionally, the timeline serves as a communication tool that aligns the team by providing a clear visual representation of the schedule and goals. Effective communication of the timeline to all team members and the utilization of project management software like Microsoft Project, Microsoft Planner, Jira or Trello can help align expectations and facilitate ongoing cooperation [2,31]. This shared understanding promotes collaboration and ensures everyone works towards common goals. Regular reviews and updates of the timeline accommodate changes and unforeseen challenges, ensuring it remains realistic and achievable [31]. By following these guidelines, project managers can create detailed and realistic schedules that drive their projects toward successful completion.

### Testing

Once the design phase is complete, the project team begins a series of experiments designated for each team member, ensuring that proper control experiments are performed to validate the results. At this stage of the project, any changes in the experimental approach should be consulted with the project sponsor and/or project manager to ensure they meet the requirements and specifications selected during the design phase. Accurate documentation of experimental procedures and results is critical for maintaining data integrity and transparency. This includes not only data and uses methodologies but also any troubleshooting steps selected to improve existing protocols. Documentation and data management practices should be consistently applied between members of the project team with oversight by the project lead and primary investigator [19,21, 34].

### Data analysis

The data generated during the testing phase should be analysed using the best available methods and analytic software. The selection of the proper statistical analysis method is especially crucial to evaluate the quality and significance of the results. Statistics and data analysis are vital skills that include understanding descriptive statistics, hypothesis testing, regression analysis, ANOVA, statistical software proficiency (like R or Python) and data visualization techniques. In addition, the team must interpret complex datasets to draw meaningful conclusions from their research experiments, all while considering the specific statistical challenges related to their field of study [34]. Analysis of data quality is an extremely important step before moving on to the final stage of the project [34].

### Conclusion

Once testing and data analysis are complete and the project results are acceptable for all participating researchers, the results are summarized, interpreted and communicated in the form of a manuscript. Results should be contextualized into the bigger research picture, and directions for future research questions should be articulated.

Open Access practices should be considered during the conclusion phase. To verify the quality of research and ensure transparency and reproducibility of the experiments, all methods used should be described coherently and in detail. No less important is the proper storage and organization of all generated data as well as preservation of biological samples, such as microbial isolates, DNA extracts or unique and limited reagents. These materials should be catalogued and stored under the most optimal conditions following institutional and product standards to ensure their integrity over a long period.

## Alternative project management approaches

Depending on the project’s needs, laboratory management in the microbial sciences may benefit from alternative approaches, including methodologies such as Six Sigma or the Critical Path Method. The Six Sigma method has been successfully applied in the healthcare and pharmaceutical manufacturing industries, emphasizing the generation of high-quality products through a data-driven approach that reduces variability while constantly improving quality and maximizing efficiency [35,38]. The Six Sigma approach is best suited for manufacturing-related and repetitive projects, where its focus on process optimization, error reduction and quality control can be fully utilized. The proper implementation of Six Sigma methodology in healthcare services often results in cost reductions, a significant decrease in the incidence of complications and shorter waiting times [37]. Its implementation in microbiological laboratories is most effective in routine laboratory testing, method validation or industrial microbiology, where consistency and precision are crucial [39,40].

The critical path method (CPM) relies on identifying and prioritizing key milestones and specific tasks necessary to complete the project, as well as developing an accurate timeline for completion [41]. This methodology is frequently applied to construction projects, in which multiple independent tasks crucial for completion must be performed according to a specific schedule. CPM is also recommended for enhancing management in clinical areas and improving drug and biomedical device development, as it focuses on evidence-based risk elimination and quality improvement [41,43]. In the laboratory, CPM can be implemented for well-defined projects with established protocols to optimize efficiency and improve standards of good practice [41]. A critical path method can also be successfully utilized as a tool to analyse scientific productivity beyond the research laboratory [42,44]. Identification and then adequate addressing of research problems and bottlenecks can lead to a substantial increase in creativity and productivity of researchers [44,45].

## Risks and challenges in project development

Several factors may lead to project failure in research laboratories. Identifying potential risks and proper prevention are key steps during the early stages of project planning [46,47]. Insufficient evaluation of laboratory risks also endangers the safety of employees, compromises the integrity and reproducibility of the data and ensures that the projects are completed in the most rigorous and resource-conscious way possible [48]. While it is impossible to outline all risks associated with laboratory projects, many tools and techniques are available for investigators to identify and assess risk [48].

Some of the most common problems include the following:

### Poor planning

Insufficient or inadequate planning can lead to project failure [49]. This may include failing to clearly define project goals, allocate and procure resources, identify critical experimental controls or create a realistic project schedule [47]. A successful research project results in the production of reliable and reproducible data and, therefore, requires the inclusion of proper quality measures at very early stages of the project [13]. In a microbiology laboratory, poor planning and disorganization of work may increase the risk of contamination of tested samples. This problem can jeopardize the progress of the experiment [40]. General disorganization and poor planning may result in inefficient work progress, wasted time and resources, frustration and, consequently, burnout of the individuals involved in the project [50,51].

### Inadequate resources

Lack of funding, personnel or equipment can obstruct a project’s progress, leading to delays and potential failure. During project planning, underestimating the time and money required for the completion of the project may result in significant delay or early termination of the research [52].

### Technical challenges

Complex or poorly understood scientific concepts, inadequate or faulty equipment, untrained project participants or unexpected results can all create significant challenges to the success of a project and potentially endanger laboratory personnel [53]. The implementation of user training procedures will improve the reproducibility and reliability of results, and routine maintenance will reduce the downtime of critical equipment. Inconsistent reagents and commercially available kits can cause significant delays due to troubleshooting and assay re-optimization. A thorough understanding of experimental procedures (including knowledge of underlying biological and chemical principles) and the inclusion of reliability controls are critical for the efficient evaluation and subsequent adjustment to the protocol. The proper execution of the project requires a good understanding of safety guidelines and the utilization of the appropriate work skills as outlined by the project manager/primary investigator (PI) and institution [53,55].

### Poor data management

Data safety and availability are a very important part of project management in research laboratories, where the integrity of data and intellectual property can directly impact the validity of scientific outcomes. Additionally, experimental data may provide support for patent applications; it is essential to prevent premature disclosure of results that could compromise the application process and lead to conflict about intellectual property rights.

In addition to the traditional physical version of lab notebooks, researchers often choose Electronic Lab Notebooks (ELNs) or Laboratory Information Management Systems such as Benchling, Labguru, Lab Archives or OneNote. ELNs are significantly beneficial for facilitating communication between team members by being easily shareable and searchable. However, extreme care must be taken to ensure that data integrity is not lost because of outdated software, digital manipulation, security breach or digital platform disruption (downtime or shutdown). Long-term storage of the data and results can be addressed by using external hard drives or cloud services like Amazon Web Services, Google Cloud or Microsoft Azure, supported with automated backup systems and secure local servers. Proper data management practices protect against frustrating software or hardware failures that could lead to permanent data loss, ensuring that research findings remain intact and available. When selecting an ELN, consideration must be given to safeguarding privacy and protected health information for projects involving patient samples and/or human subjects.

### Communication breakdown

Poor communication among team members can lead to misunderstandings, delays and mistakes, which can ultimately lead to project failure [8]. It is important to clearly define roles and tasks for each member of the team and choose the person responsible for conducting each step of the project. Depending on the scale of the project, regular team meetings provide a great opportunity to navigate the project and discuss ongoing challenges and changes, and the frequency and duration of the meetings should be adjusted to meet actual progress to maximize efficiency and productivity and to avoid unnecessary loss of time [56]. Development of a standard operating procedure is recommended for communicating expectations, laboratory policies and other critical information required for efficient management of the laboratory [57,58]. A complex research project often requires collaboration between different laboratories and institutions. Therefore, navigation of tasks and interpersonal communication create challenges that must be closely monitored and adjusted to avoid misunderstanding and miscommunication with external project partners [59].

### Changes in a project or a lack of a defined scope

Changes in the scope of a project can lead to additional work, delays and increased costs, which may make it difficult to achieve project goals [15,47]. Research projects can become extremely complicated tasks, based on multiple assumptions and unclear data [49]. Adapting the research goals to the newly acquired data is essential for the progress of research. Still, constant changes of the project scope or research methodology may lead to serious delays or permanent inhibition of progress [47,60].

### Health and safety hazards

Research laboratories, specifically microbiology-focused laboratories, create an environment where researchers are exposed to various biological, chemical and physical factors that may be a cause of accidents potentially hazardous to human health. These accidents can occur from direct contact of the infectious agent and corrosive chemicals with the mucous membranes of the eyes, nose or mouth. Depending on the nature of work, microbiological research often involves work with various pathogenic strains of viruses, bacteria, fungi and parasites. Proper training, personal protective equipment and adequate sites for work with pathogens are essential to minimize chances of contamination or accidental infection of the lab personnel [40,54, 55]. In addition to biological infectious materials, various experimental procedures, like genetic manipulation, protein or nucleic acid isolation or further molecular and biochemical modification of cells and cell products, require the utilization of corrosive, mutagenic or carcinogenic substances. Improper handling and storage of those materials may cause devastating consequences for the health and safety of researchers [54,55, 61]. A general understanding of safety rules and identification of potential biological and chemical risks is essential for proper functioning in the research environment [53,54]. Laboratory safety standards and protocols should be established in collaboration with the institution’s Office of Health and Safety. These must be communicated to all members of the research team, with updated manuals available in the laboratory for quick reference.

### External factors

External factors, such as regulatory changes or unexpected events like natural disasters or pandemics, can also disrupt a project’s progress and potentially lead to project failure [62]. Restrictions implemented due to the Coronavirus disease (COVID-19) pandemic greatly impacted research facilities, compromising the efficiency of the research and training programmes [63,65]. Proper research progress tracking and adequate data storage can help mitigate the consequences of the abrupt project break. To increase the chances of accomplishing predefined objectives, it is essential to detect these potential factors early in a project and to proactively manage and mitigate them throughout the period in which research activities are conducted [50]. Effective project management and regular communication among team members can also help identify and address potential issues before they become significant problems.

## Building an effective and collaborative laboratory team

**Fig. 2.**
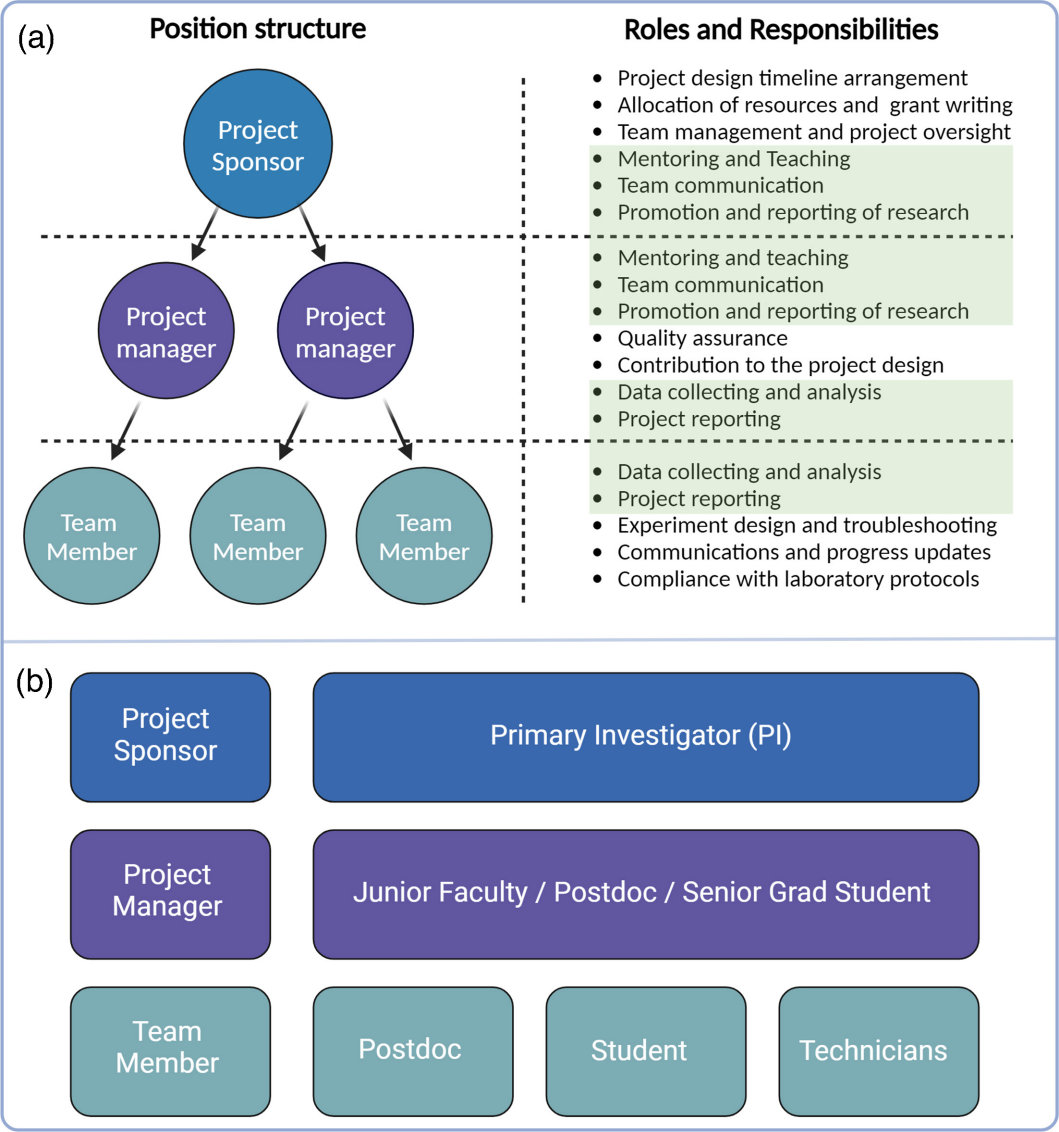
Organizational structure and roles of a laboratory research team. (**a**) A research team is composed of the main stakeholder/project sponsor who determines the general scope of the project, the project manager who ensures the progress of the research and other team members responsible for their respective tasks. Task designations for each team member contain unique and shared roles and responsibilities. Overlapping roles are highlighted in green. (**b**) In laboratory settings, the primary investigator typically assumes the role of the project sponsor, while project managers can be assigned to junior faculty, postdocs or graduate students. Additional team members are selected based on their respective skills and research experience.

Designing the project plan requires careful analysis of available resources, effective team management and a general understanding of the technical challenges. Establishing a cooperative team structure is essential for effective project planning, the formation of research hypotheses and the execution of research objectives [66,67]. This step is an essential part of defining roles and responsibilities, organizing the team and coordinating team members to their respective tasks [68]. Proper distribution of tasks to individual team members should be based on their skills, experience and professional ambitions, which in exchange can significantly improve productivity, morale and resource utilization, leading to a more refined experimental design [31,68]. The ever-changing world of science research and development requires the cooperative work of numerous people, which implies the necessity for the structuring of the research groups [69]. Well-managed research teams are composed of a main stakeholder/project sponsor who determines the general scope of the project, the project manager who ensures the progress of the research and other team members responsible for their respective tasks (Fig. 2a). A cooperative environment is strengthened by the fair distribution of roles and responsibilities among research team members (Fig. 2b).

### Project sponsor

Typically referred to as the PI in laboratory settings, the central role of the project sponsor is to navigate the project inauguration and progress. That task requires the establishment of the project timeline and the assembly of the research team. In addition, the project sponsor is responsible for providing adequate research allocation via grant management or other means of fiscal support. In collaboration with project managers and team members, the project sponsor oversees the preparation and writing of manuscripts, ensuring that data are appropriately analysed and interpreted, protocols are accurately outlined and appropriate recognition is given to contributors.

Beyond their role in overseeing scientific progress, project sponsors should also have a good understanding of effective leadership and organizational practices. Effective teams thrive when project sponsors communicate their vision to inspire their team, demonstrate empathy, prioritize integrity, encourage diverse perspectives and develop strong team values [70]. Importantly, cultivating a healthy and supportive laboratory environment yields a motivated team that will function and collaborate optimally. An effective project sponsor builds trust and psychological safety with their team members so that individuals are comfortable bringing new ideas and perspectives to the project, ultimately resulting in a cohesive, unified scientific team [71].

### Project manager

The core competency of a project manager lies in designing and overseeing key steps for work advancement, quality assurance and proper documentation of each experiment and methodology used to obtain the data [72,73]. Accurate project reports and data analysis are essential to maintain the level of reproducibility and accountability required for high-quality research [74,76]. The remaining daily project tasks are performed by the project manager and/or additional team members, each assigned a specific role. The project manager facilitates recurring team meetings to ensure that communication between team members is assured. The roles of the project sponsor and project manager may overlap in the process of mentorship and team management, which are necessary to ensure the progress of the research [77]. The project manager may also be tasked with directly training team members and ensuring that all safety protocols are followed [78]. In laboratory research environments, the project manager role may be assumed by a junior faculty member, postdoctoral fellow or senior graduate student [79].

### Team member(s)

Laboratory team members are tasked with implementing the experimental plan according to the plan designed by the project sponsor and project manager. In addition to performing well-controlled experiments and collecting data, laboratory team members may be required to troubleshoot and optimize experimental protocols, maintain instrumentation, generate needed reagents and/or seek additional specialized training necessary to execute the experimental plan. Tasks should be assigned to team members that promote collaboration and synergy between personnel, and competing efforts should be avoided. Team members also maintain detailed experimental protocols to ensure that methodologies are accurately communicated to other current and future team members. Less experienced postdoctoral fellows, students and technicians typically constitute the laboratory team within a research laboratory context.

## Impact of team size on project planning

The size and experience of the laboratory or group collaborating on a particular project can have a noteworthy impact on the selection of the project management approach [80]. Different project management approaches have different strengths and weaknesses, and the size of the laboratory or research group can influence how effective each approach will be [13]. For smaller laboratories or teams, agile project management may be the most effective approach [81]. Agile methodology is highly collaborative and relies on frequent communication and feedback between team members. In smaller teams, it is easier to maintain a frequent number of meetings or casual interactions within the laboratory, allowing for a better flow of information among team members and making agile methodology a good fit [22,81, 82]. For larger laboratories or teams, a hybrid approach may be more effective. Large teams may benefit from the structure and guidance provided by the waterfall approach for certain phases of the project but also need the flexibility and adaptability of agile methodology for other phases. Teams may customize their course of action by adopting a hybrid approach that fits precisely to the group-specific and project-specific requirements.

## Conclusion

As a rule, the choice of project configuration should be customized and adapted following the specific needs of the laboratory or team to ensure the most effective and efficient project management possible. By utilizing the appropriate methodology, teams in microbiological and biomedical laboratories can improve productivity and increase the chances of success for their projects. A flexible and adaptable approach that allows adjustments as needed will help ensure the project stays on track and meets its goals and objectives. Effective team communication and time management are essential competencies required for laboratory teams to achieve synergy toward achieving their research objectives. The successful implementation of project and team management strategies into the microbial research laboratory will enhance organization, improve resource allocation and streamline communication, ultimately leading to more efficient and productive research outcomes.

## Abbreviations

CPM: critical path method
ELNs: Electronic Lab Notebooks
PI: primary investigator

## References

[1] Dhir SK, Gupta P (2021). Formulation of research question and composing study outcomes and objectives. Indian Pediatr.

[2] Stempfle J, Badke-Schaub P (2002). Thinking in design teams - an analysis of team communication. Design Studies.

[3] Hall NG (2012). Project management: Recent developments and research opportunities. J Syst Sci Syst Eng.

[4] White D, Fortune J (2002). Current practice in project management — an empirical study. Int J Project Manag.

[5] Lenfle S (2008). Exploration and project management. Int J Project Manag.

[6] Bongiovanni A, Colotti G, Liguori GL, Di Carlo M, Digilio FA (2015). Applying quality and project management methodologies in biomedical research laboratories: a public research network’s case study. *Accred Qual Assur*.

[7] Dorwal P, Sachdev R, Gautam D, Jain D, Sharma P (2016). Role of whatsapp messenger in the laboratory management system: a boon to communication. J Med Syst.

[8] Gorse CA, Emmitt S (2007). Communication behaviour during management and design team meetings: a comparison of group interaction. Construct Manag Econ.

[9] Milojević S (2014). Principles of scientific research team formation and evolution. Proc Natl Acad Sci U S A.

[10] Payne JM, France KE, Henley N, D’Antoine HA, Bartu AE (2011). Researchers’ experience with project management in health and medical research: results from a post-project review. BMC Public Health.

[11] Pacheco Junior MA, Anholon R, Rampasso IS, Leal Filho W (2020). Improving research labs’ performance through project management guidelines: a case study analysis. Int J Product Perf Mgmt.

[12] Artto K, Kujala J, Dietrich P, Martinsuo M (2008). What is project strategy?. Int J Project Manag.

[13] Brocke J vom, Lippe S (2015). Managing collaborative research projects: a synthesis of project management literature and directives for future research. Int J Project Manag.

[14] König B, Diehl K, Tscherning K, Helming K (2013). A framework for structuring interdisciplinary research management. Research Policy.

[15] Marnada P, Raharjo T, Hardian B, Prasetyo A (2022). Agile project management challenge in handling scope and change: a systematic literature review. Proc Comput Sci.

[16] Säisä M, Tiura K, Janne R (2018). Waterfall vs. agile project management methods in university-industry collaboration projects.

[17] Quist C (2015). Benefits of blending agile and waterfall project planning methodologies.

[18] Jinzenji K, Jin A, Muramoto T (2020). Productivity evaluation indicators based on LEAN and their application to compare agile and waterfall projects.

[19] Boyd JC, Rifai N, Annesley TM (2009). Preparation of manuscripts for publication: improving your chances for success. Clin Chem.

[20] Fernandes G, Moreira S, Araújo M, Pinto EB, Machado RJ (2018). Project management practices for collaborative university-industry R&D: a hybrid approach. Proc Comput Sci.

[21] Aguinis H, Hill NS, Bailey JR (2021). Best practices in data collection and preparation: recommendations for reviewers, editors, and authors. Organ Res Methods.

[22] Denning S (2016). Understanding the three laws of agile. Strat Lead.

[23] Galli BJ (2021). The value of communication in agile project management. Int J Strat Eng.

[24] Papadakis E, Tsironis L (2020). Towards a hybrid project management framework: a systematic literature review on traditional, agile and hybrid techniques. J Modern Project Manag.

[25] Tatikonda MV, Rosenthal SR (2000). Successful execution of product development projects: balancing firmness and flexibility in the innovation process. J Ops Management.

[26] Ismail R (2017). Hybrid project management: agile with discipline.

[27] Copola Azenha F, Aparecida Reis D, Leme Fleury A (2020). The role and characteristics of hybrid approaches to project management in the development of technology-based products and services. Proj Mgmt Jrnl.

[28] Lesmana IPD, Karimah RN, Widiawan B (2016). Agile-Waterfall hybrid for prevention information system of dengue viral infections: a case study in Health Department of Jember, East Java, Indonesia.

[29] Willis LD (2023). Formulating the research question and framing the hypothesis. Respir Care.

[30] Covvey JR, McClendon C, Gionfriddo MR (2024). Back to the basics: Guidance for formulating good research questions. Res Soc Adm Pharm.

[31] Girard P, Robin V (2006). Analysis of collaboration for project design management. Comput Indust.

[32] Irfan M, Khan SZ, Hassan N, Hassan M, Habib M (2021). Role of project planning and project manager competencies on public sector project success. Sustainability.

[33] Sunmola HO (2020). Evaluation of motivating and requiring factors for milestones in IT projects. Procedia Manuf.

[34] Casadevall A, Ellis LM, Davies EW, McFall-Ngai M, Fang FC (2016). A framework for improving the quality of research in the biological sciences. mBio.

[35] Byrne B, McDermott O, Noonan J (2021). Applying lean six sigma methodology to a pharmaceutical manufacturing facility: A case study. *Processes*.

[36] Bell E, Vossen B, Tobin E, Gerdes D, Mahmoud M (2023). The efficiency of six sigma in the pharmaceutical industry.

[37] Niñerola A, Sánchez-Rebull M-V, Hernández-Lara A-B (2020). Quality improvement in healthcare: six Sigma systematic review. Health Policy.

[38] Kam AW, Collins S, Park T, Mihail M, Stanaway FF (2021). Using lean six sigma techniques to improve efficiency in outpatient ophthalmology clinics. BMC Health Serv Res.

[39] Inal TC, Goruroglu Ozturk O, Kibar F, Cetiner S, Matyar S (2018). Lean six sigma methodologies improve clinical laboratory efficiency and reduce turnaround times. J Clin Lab Anal.

[40] Carey RB, Bhattacharyya S, Kehl SC, Matukas LM, Pentella MA (2018). Practical guidance for clinical microbiology laboratories: Implementing a quality management system in the medical microbiology laboratory. Clin Microbiol Rev.

[41] Kelley JE, Walker MR (1959). Critical-path planning and scheduling.

[42] Hofmann PA (1993). Critical path method: an important tool for coordinating clinical care. Jt Comm J Qual Improv.

[43] Godderis L, Vanhaecht K, Masschelein R, Sermeus W, Veulemans H (2004). Prevention pathways: application of the critical path methodology in occupational health services. J Occup Environ Med.

[44] Loehle C (1994). A critical path analysis of scientific productivity. J Creat Behav.

[45] Huber JC (1998). Invention and Inventivity as a special kind of creativity, with implications for general creativity. J Creat Behav.

[46] Munns AK, Bjeirmi BF (1996). The role of project management in achieving project success. Int J Project Manag.

[47] Mirza MN, Pourzolfaghar Z, Shahnazari M (2013). Significance of scope in project success. Procedia Technol.

[48] Tziakou E, Fragkaki AG, Platis AΝ (2023). Identifying risk management challenges in laboratories. *Accred Qual Assur*.

[49] Tress B, Tress G, Fry G (2005). Researchers’ experiences, positive and negative, in integrative landscape projects. Environ Manage.

[50] Gällstedt M (2003). Working conditions in projects: perceptions of stress and motivation among project team members and project managers. Int J Project Manag.

[51] Maslach C, Leiter MP (2016). Understanding the burnout experience: recent research and its implications for psychiatry. World Psychiatry.

[52] Lippi G, Mattiuzzi C (2019). Project management in laboratory medicine. J Med Biochem.

[53] Bruns HC (2009). Leveraging functionality in safety routines: Examining the divergence of rules and performance. *Human Relations*.

[54] Ellis JG, Riches NJ (1978). Safety in Biology Laboratories. Safety and Laboratory Practice.

[55] Peichl P (2000). Health, safety and environmental protection in a biological research laboratory. Int Arch Occup Environ Health.

[56] Roels G, Corbett CJ (2021). Too many meetings? Scheduling rules for team coordination. SSRN J.

[57] Ezzelle J, Rodriguez-Chavez IR, Darden JM, Stirewalt M, Kunwar N (2008). Guidelines on good clinical laboratory practice: bridging operations between research and clinical research laboratories. J Pharm Biomed Anal.

[58] Gilligan PH (2004). Impact of clinical practice guidelines on the clinical microbiology laboratory. J Clin Microbiol.

[59] Numprasertchai S, Igel B (2005). Managing knowledge through collaboration: multiple case studies of managing research in university laboratories in Thailand. *Technovation*.

[60] McElroy W (1996). Implementing strategic change through projects. Int J Project Manag.

[61] Leggett DJ (2012). Identifying hazards in the chemical research laboratory. Proc Safety Prog.

[62] Luo Y, Wang J, Zhang M, Wang Q, Chen R (2021). COVID-19-another influential event impacts on laboratory medicine management. J Clin Lab Anal.

[63] Termini CM, Traver D (2020). Impact of COVID-19 on early career scientists: an optimistic guide for the future. BMC Biol.

[64] Haleem A, Javaid M, Vaishya R, Deshmukh SG (2020). Areas of academic research with the impact of COVID-19. Am J Emerg Med.

[65] Stenson MC, Fleming JK, Johnson SL, Caputo JL, Spillios KE (2022). Impact of COVID-19 on access to laboratories and human participants: exercise science faculty perspectives. Adv Physiol Educ.

[66] Nierse CJ, Schipper K, van Zadelhoff E, van de Griendt J, Abma TA (2012). Collaboration and co-ownership in research: dynamics and dialogues between patient research partners and professional researchers in a research team. Health Expect.

[67] Katz JS, Martin BR (1997). What is research collaboration?. Res Policy.

[68] Byron K, Keem S, Darden T, Shalley CE, Zhou J (2023). Building blocks of idea generation and implementation in teams: a meta‐analysis of team design and team creativity and innovation. Pers Psychol.

[69] Kyvik S, Reymert I (2017). Research collaboration in groups and networks: differences across academic fields. Scientometrics.

[70] Robel S (2022). Building leadership skills in research groups. *Curr Protoc*.

[71] Brown S-A, Sparapani R, Osinski K, Zhang J, Blessing J (2023). Team principles for successful interdisciplinary research teams. *Am Heart J Plus*.

[72] Duhovnik J, Tavčar J, Koporec J (1993). Project manager with quality assurance. Comput Aided Design.

[73] Chipulu M, Neoh JG, Ojiako U, Williams T (2013). A Multidimensional Analysis of Project Manager Competences. IEEE Trans Eng Manage.

[74] Ford M (2008). Disciplinary authority and accountability in scientific practice and learning. Sci Ed.

[75] Winsberg E, Huebner B, Kukla R (2014). Accountability and values in radically collaborative research. Stud Hist Philos Sci.

[76] Ladd JM, Lappé MD, McCormick JB, Boyce AM, Cho MK (2009). The “how” and “whys” of research: life scientists’ views of accountability. J Med Ethics.

[77] Sewchurran K, Barron M (2008). An investigation into successfully managing and sustaining the project sponsor—Project manager relationship using soft systems methodology. Project Manag J.

[78] Ritterson R, Casagrande R (2017). Basic scholarship in biosafety is critically needed to reduce risk of laboratory accidents. *mSphere*.

[79] Munson E, Bowles EJ, Dern R, Beck E, Podzorski RP (2018). Laboratory focus on improving the culture of biosafety: Statewide risk assessment of clinical laboratories that process specimens for microbiologic analysis. J Clin Microbiol.

[80] Fried L (1991). Team size and productivity in systems development bigger does not always mean better. J Inform Syst Manag.

[81] de O. Melo C, S. Cruzes D, Kon F, Conradi R (2013). Interpretative case studies on agile team productivity and management. Inform Softw Technol.

[82] Lee S, Yong H-S (2013). Agile software development framework in a small project environment. J Informa Process Syst.

